# When do perturbative approaches accurately capture the dynamics of complex quantum systems?

**DOI:** 10.1038/srep28204

**Published:** 2016-06-23

**Authors:** Amir Fruchtman, Neill Lambert, Erik M. Gauger

**Affiliations:** 1 Department of Materials, University of Oxford, Oxford OX1 3PH, United Kingdom; 2 CEMS, RIKEN, Saitama, 351-0198, Japan; 3 SUPA, Institute of Photonics and Quantum Sciences, Heriot-Watt University, EH14 4AS, United Kingdom

## Abstract

Understanding the dynamics of higher-dimensional quantum systems embedded in a complex environment remains a significant theoretical challenge. While several approaches yielding numerically converged solutions exist, these are computationally expensive and often provide only limited physical insight. Here we address the question: when do more intuitive and simpler-to-compute second-order perturbative approaches provide adequate accuracy? We develop a simple analytical criterion and verify its validity for the case of the much-studied FMO dynamics as well as the canonical spin-boson model.

Recent years have seen remarkable experimental progress in probing and controlling increasingly larger quantum systems in condensed matter systems^1^,^2^,^3^. In this setting it is often not possible to consider the environment as only having a very small perturbative influence on the system of interest. Therefore, one cannot a priori expect conventional weak-coupling approaches^4^, attractive for their relative simplicity and interpretability, to remain a suitable tool for these systems. However, master equations (MEs) based on a perturbative expansion nevertheless often provide useful solutions in a variety of circumstances. This includes, for example, comparison of such a model to experimentally observed excition-induced dephasing for laser-driven Rabi oscillation in semiconductor quantum dots^5^,^6^, or the close agreement of ME models with numerically exact solutions^7^,^8^,^9^. In some but not all of those cases, a judiciously chosen transformation allows a redefinition of system and environment before the perturbative expansion is performed (see, e.g. refs 7 and 8), allowing for better performance.

An important question to address then is: when is an approach that is perturbative to second order in the coupling strength ‘good enough’ for capturing the essentials of the dynamics on a qualitative, or even quantitative, level? Here, we develop a criterion for predicting when such an approach is expected to perform well. Our criterion is based on a reasonably straightforward analytical expression that is, crucially, easy to evaluate, whilst also lending itself to an intuitive physical interpretation.

We wish to stress that we are focussing on the question when a second-order perturbative treatment can be used reliably, as opposed to when environmental effects only perturb the system slightly. In the latter case the perturbative treatment should be automatically valid, however, a second-order treatment may remain valid even when it changes the dynamics dramatically from those of an isolated system. Hereafter, we shall use the term ‘weak-coupling’ synonymously with ‘perturbative to second-order’.

We consider two different weak-coupling techniques: time convolutionless (TCL) master equations^4^ and a second method based on the phase-space representation of the full density matrix^10^ (P-mat). Interestingly, we show that both approaches give rise to exactly the same criterion, despite their rather different nature. This suggests that our criterion has applicability beyond just one particular perturbative approach.

We apply both approaches to the canonical spin boson model^11^ as well as the much studied FMO complex^12^,^13^,^14^,^15^,^16^,^17^. The latter has received a significant amount of attention and is a prime example of the complicated interplay between coherent dynamics interwoven with significant environmental influences. The advantage of this system is that a large body of literature and numerically converging methods exist. Interestingly, our criterion indicates that despite the relatively strong coupling, a second-order treatment is appropriate at lower but not necessarily at higher temperatures. We note that the FMO problem has previously been tackled with weak-coupling techniques^9^,^15^,^18^,^19^,^20^,^21^, but here we not only use a novel method but also introduce a rigorous criterion for when such approaches are indeed permissible.

In the ‘grey area’ where a second-order expansion is no longer strictly justified, we find that the quality of the different approaches differs. Some of us have previously found that the P-mat method outperformed the commonly used secular, second-order Born-Markov master equation^10^. For the examples studied here, we find that a TCL ME gives slightly better short time dynamics than P-mat, whilst the latter frequently performs better at longer times as the system approaches thermalisation. As expected, fourth-order TCL typically (but not always) beats second-order approaches, but may also lead to unphysical results in the strong-coupling regime. To arrive at robust conclusions, we supplement comparisons of the population dynamics (as in refs 9 and 22) by the trace distance, which is also sensitive to the agreement between the coherences of the approximate and exact methods.

## Results

As a starting point we use the ‘standard’ open-systems Hamiltonian, which is given by


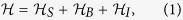


where 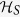 is a finite-dimensional Hamiltonian for the system of interest, and 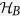, 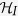 are the bath and interaction Hamiltonians, respectively. The bath (or baths in the case of the FMO complex) is modelled by a collection of harmonic oscillators that are linearly coupled to the system according to:


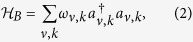



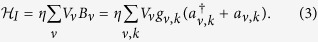


Here, *V*_*ν*_ are system and *B*_*ν*_ the bath operators with bath index *ν*. The *g*_*ν*,*k*_ are the coupling constants (in units of energy), and 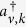 the bosonic creation operators satisfying 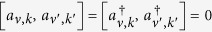, 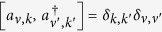.

Note that for the case of phonons, the justification for the linear form of (3) usually hinges on a weak-coupling argument: We assume that the equilibrium position of the atoms hosting the vibrational modes only vary slightly when an electron or an exciton is present. Expanding the potential energy between these atoms to first order leads to the linear coupling term of Eq. (3)^11^,^23^. Conversely, for very strong coupling, one would not be able to justify the first-order approximation.

The canonical definition of the coupling strength of an open system to its environment is the reorganization energy, i.e. the potential energy associated with shifting the oscillator modes into their new equilibrium position in the presence of an excitation of the system,


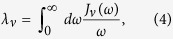


where 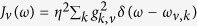.

Weak-coupling is sometimes defined by the condition 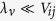 where *V*_*ij*_ represent off-diagonal coupling elements of the Hamiltonian between distinct basis states (typically chosen as the site basis)^13^,^24^. Whilst straightforward for a two level system, in higher-dimensional systems such as the FMO complex, one may wonder exactly which *V*_*ij*_ ought to be considered. Arguably, some off-diagonal terms in the Hamiltonian may be small or even vanish without automatically implying a strong coupling scenario. The authors of ref. 22 write that in many cases the “ … reorganization energy is not a reasonable measure for the coupling strength …” because the system’s dynamic frequencies are not taken into consideration. They suggest circumventing this shortcoming by defining an effective reorganisation energy 
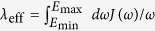
, which only spans the relevant energy interval containing the system frequencies. On the other hand, in ref. 25 the authors present a heuristic approach for estimating when a weak-coupling approach is valid by introducing a measure that depends on the temperature of the bath, but in that case not on the frequencies of the system.

In this paper we analyse a different approach, which takes into account both the system frequencies and the temperature of the environment. Essentially, we let ourselves be guided by wishing to apply the term “weak-coupling” to situations when higher order expansion terms beyond the second order are not required for reliably capturing the open systems dynamics. To this end, we explicitly compare terms from a 4^th^ order expansion to 2^nd^ order terms, obtaining the following weak-coupling criterion:


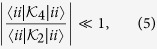


which must hold separately for each eigenenergy *i*, and where 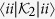 and 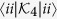 are explicitly given in Eqs (29) and (31) of the methods section, and are rates given by TCL2 and TCL4 perturbation expansion. This “full” criterion, whilst evaluated straightforwardly enough, does not easily lend itself to providing much analytical insight. Therefore, we also consider a “simplified” version that is more amenable to physical interpretation. This simplified criterion reads


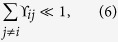


where


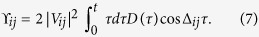


Here, 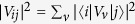 with |*j*〉 being the eigenstates of the system, Δ_*ij*_ = *ε*_*i*_ − *ε*_*j*_ is the energy difference between two eigenenergies *i*, *j*, and we have assumed that all spectral densities take the same form *J*_*ν*_(*ω*) = *J*(*ω*). (Generalising for different spectral densities is straightforward.) For a thermal environment we have 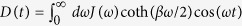. Further, *t* is the timescale of interest (i.e. the duration for which we want the calculations to remain accurate). When *D*(*τ* > *t*) ≈ 0, it is justified to take the upper limit of the integral to infinity. We provide the explicit derivation of Eq. (6) in the ‘Derivation of the Criterion’ section, and note that TCL and P-mat both lead to exactly the same final expression.

For the examples studied in the following sections of this paper, the simplified criterion of Eq. (6) turns out to be as stringent as the full criterion. However, not having been able to mathematically prove that it will always be sufficiently rigorous, we suggest using it carefully and supplementing it by Eq. (5) if in doubt.

Interestingly, the quantities written in the RHS of Eq. (7) are exactly the terms leading to the “slippage of initial conditions”^26^,^27^, explained below, indicating that if the initial slippage for each eigenstate is small, then the perturbative treatment is a good approximation, otherwise one should look for alternative methods. Note that appreciable slippage of initial conditions marks the onset of non-Markovian effects, rendering a perturbative second-order expansion (and thus our definition of weak coupling) invalid.

Let us briefly explain what the slippage of initial conditions is: This is a second-order argument, and is related to the relationship between the Redfield master equation and the TCL2 master equation (that is discussed in more detail below): In the Schrödinger picture, the TCL2 kernel 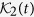 becomes constant for times larger than the memory time of the environment *τ*_*b*_. This means that after 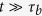 the dynamics become Markovian. In fact, making the substitution 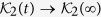 yields exactly the (Markovian) Redfield equations for the dynamics of the system. The slippage of initial conditions is a mathematical trick that enables one to recover the long-time TCL2 dynamics of the system using Redfield equations, by introducing a modified initial density matrix *ρ*(0) → *ρ*_effective_(0), that captures the difference in the initial dynamics between time-dependent TCL2 and Redfield. That is, after an initial transient and non-Markovian period, the density matrix calculated using TCL2 will be similar to one calculated using Redfield starting from a modified initial state. This “initial slippage” also guarantees the positivity of the density matrix, which is not true in general for the Redfield equations.

In the methods section we introduce the perturbative methods we use in this paper, namely the TCL and P-mat methods. We also briefly introduce HEOM, a technique yielding numerically converged results, which will in the following serve as benchmark against which we may compare our perturbative expansions, and provide evidence for the validity and usefulness of the above criteria.

### Spin-Boson Model Example

As a first case study, we apply our criteria to the canonical example of the spin-boson model^11^ with Hamiltonian given by:


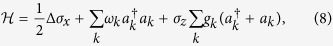


where *σ*_*x*_, *σ*_*z*_ are the usual Pauli matrices and Δ represents the Rabi frequency of the spin. Further, *a*^(†)^ denotes the annihilation (creation) operator of a bosonic mode *k* with frequency *ω*_*k*_, and *g*_*k*_ are the spin-boson coupling elements. We consider an Ohmic spectral density with exponential cutoff as follows


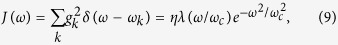


where *η* is a dimensionless parameter allowing us to easily interpolate between weak and strong coupling. We choose Δ = *π*/2 ps^−1^, *T* = 50 K, *λ* = 0.01485 ps^−1^, and *ω*_*c*_ = 2.2 ps^−1^.

**Figure 1:**
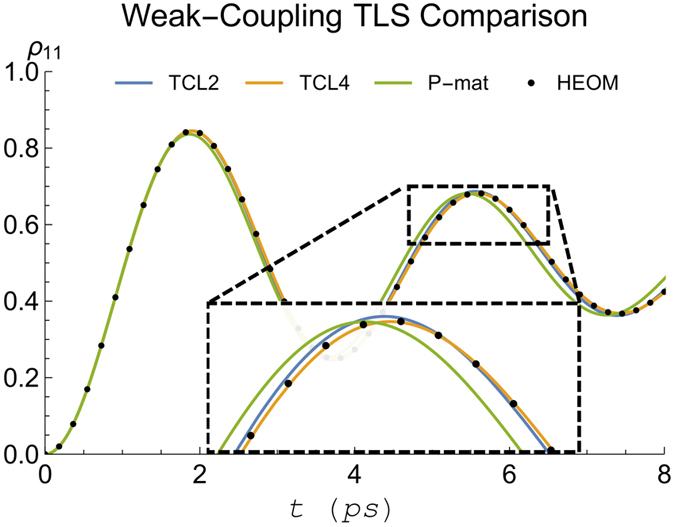
Comparison of the spin boson dynamics calculated using HEOM (dashed) to our perturbative techniques (solid). Here we consider the weak-coupling case with *η* = 1 (see main text for other parameters).

**Figure 2:**
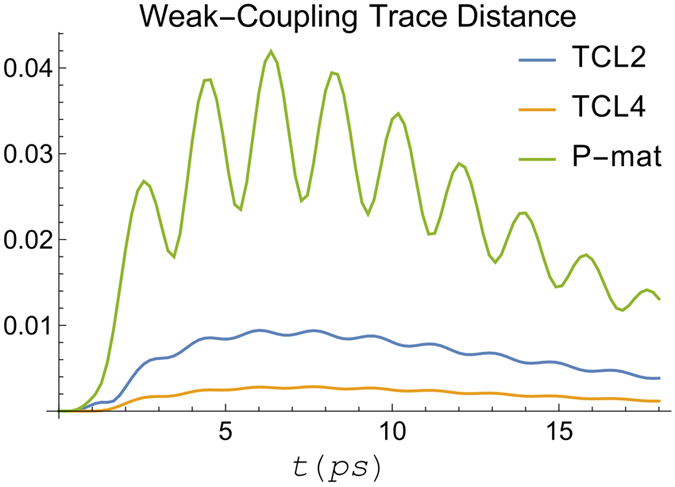
Trace distance between the spin boson dynamics calculated using HEOM and the different perturbative techniques. Again, we consider a clearly cut weak coupling scenario with parameters as in Fig. 1.

Evaluating our weak-coupling criteria, the LHS of Eqs 5 and 6 for these parameters gives 0.04 *η* (0.06 *η*) for the simplified (full) version of the criterion, meaning the system is well into the weak-coupling regime when we set *η* = 1. This can be seen in Figs 1 and 2 which show a comparison of TCL2, TCL4, and P-mat techniques with the numerically exact HEOM dynamics. As expected the perturbative techniques capture the dynamics very accurately in this case. P-mat performs worst in this case (despite having previously been shown to outperform a standard secular Born-Markov master equation in a comparable scenario^10^), although the gap narrows towards longer times when the system approaches thermal equilibrium. The trace distance of all three perturbative approaches features oscillations at twice the Rabi frequency of the natural precession time of the spin, clearly visible in Fig. 2. For completeness, the trace distance between two density matrices is defined by


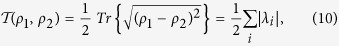


where *λ*_*i*_ is the i’th eigenvalue of (*ρ*_1_ − *ρ*_2_). Its physical meaning is connected to the probability of correctly distinguishing between the two states if a measurement was performed. For a two-level system, the trace distance is equal to 1/2 the Euclidean distance between two points in the Bloch sphere^28^. Generally, its interpretation depends on the dimensionality of the system, making it difficult to say what “small” means in absolute terms. However, this does not affect its usefulness as a relative metric for benchmarking several approximate methods against an exact solution.

**Figure 3:**
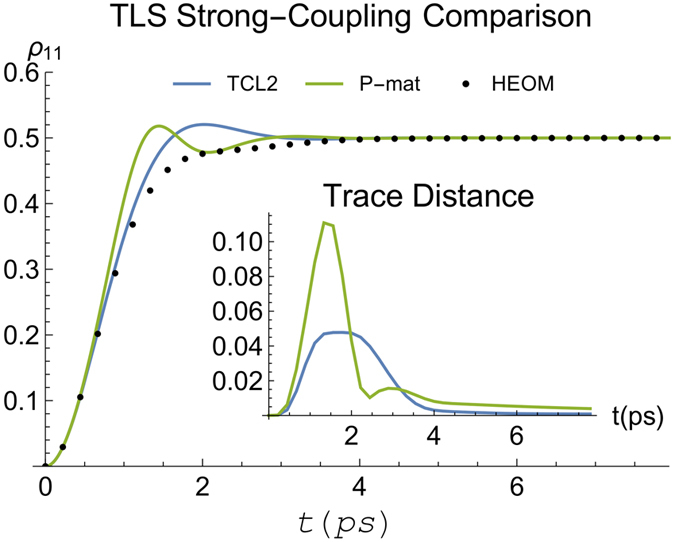
Comparison between the spin boson dynamics calculated using HEOM and weak-coupling techniques, for a strong-coupling case, *η* = 10. Other parameters as in Fig. 1.

When we increase the system and environment coupling by setting *η* = 10, our criteria indicate that we can no longer expect to be in the weak-coupling regime. In Fig. 3 we show a comparison between TCL2 and P-mat techniques with exact HEOM calculations, and it is apparent that both fail not only quantitatively but also qualitatively at intermediate times around *t* ≈ 1.5 ps. We do not show TCL4 in this case because the TCL4 generator features an unphysical positive eigenvalue in the long time limit.

### FMO Complex Dynamics Example

We now turn to the dynamics of the Fenna-Matthews-Olsen (FMO) complex – a prime example of complex quantum dynamics in the difficult regime between weak and strong environmental coupling in a higher-dimensional Hilbert space. We follow the 7-site FMO model considered by Ishizaki and Fleming^13^, where all chlorophylls have the same environment given by a Drude-Lorentz spectral density 
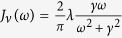
 with *λ* = 6.59 ps^−1^. We consider the three cases for the environmental parameters discussed in ref. 13, namely *T* = 77 K, *γ*^−1^ = 50 ps; *T* = 300 K, *γ*^−1^ = 50 ps; and *T* = 300 K, *γ*^−1^ = 166 fs. Once again comparing HEOM results to two perturbative methods, we will find that our criteria predict the validity of the methods not only for the spin boson model considered above but also for this much more involved case.

Table 1 lists the values of the criteria for the different sets of parameters mentioned above. Here we observe a factor of roughly two between the simplified, Eq. (6) and full criterion, Eq. (5), with the simplified version being the more stringent one. However, we note that both agree in their classifications of whether the perturbative solution should be valid or not. Further, the reorganisation energy is constant across the three cases as it does not depend on the temperature or the cut-off frequency (for this choice of spectral density), and is thus clearly not a good measure for the coupling strength in the sense discussed in this paper.

**Figure 4:**
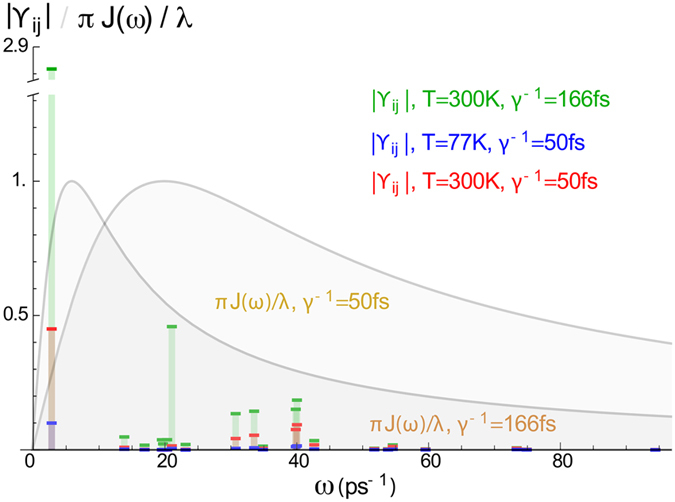
Visualisation of the simplified criterion: the different |ϒ_*ij*_| are shown at their corresponding frequencies Δ_*ij*_. The rescaled and dimensionless respective spectral densities, *πJ*(*ω*)/*ω*, are also shown as the coloured background areas, to illustrate at which frequencies environmental effects are expected to be dominant.

We visualise the ϒ_*ij*_ of Eq. (6) for all three FMO cases in Fig. 4. Notably, two of the system eigenstates are almost resonant, being split by only ~2.8 ps^−1^. This pair experiences strong environmental interactions for the *γ*^−1^ = 166 fs cutoff (ϒ_*ij*_ ~ 2.8), clearly placing the system into the strong-coupling domain, according to our definition. By contrast, for *γ*^−1^ = 50 fs this pair sits in an intermediate regime (at *T* = 300 K), whereas all other occurring frequencies satisfy our weak-coupling criterion.

Consequently, we would expect a very good agreement between exact numerics and weak-coupling techniques in the low-temperature cases at *T* = 77 K, but at *T* = 300 K we expect the weak-coupling techniques to work only for *γ*^−1^ = 50 fs and not for *γ*^−1^ = 166 fs. We shall see that these expectations are met by our dynamical simulations discussed in the following.

To perform our simulations, the real part of the response function *D*(*t*) is approximated by a single exponent and a delta function


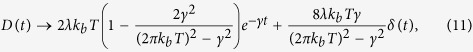


while the exact imaginary part of the response function has a single exponent. This approximation is made to overcome memory restrictions of the HEOM implantation. For a fair comparison, we use the same response function for the TCL and P-mat methods, though these methods are not restricted to a certain structure of the response function. We note that HEOM calculations for the same FMO model, but with a more accurate approximation for the response function, using of 3 (2) exponents for approximating the 77 *K* (300 *K*) response function, have been reported in ref. 29.

To capture the full density matrix dynamics as opposed to just the evolution of the populations, we shall also plot and discuss the trace-distance between the perturbative solutions and the HEOM benchmark.

**Figure 5:**
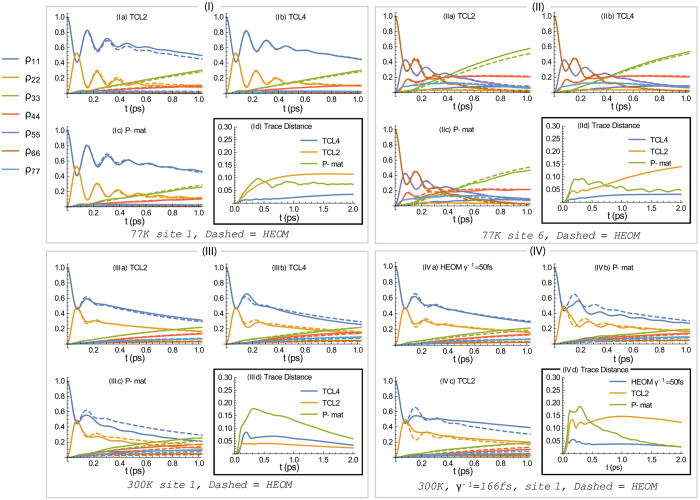
Benchmarking of dynamics obtained from perturbative techniques (solid) against numerically converged HEOM (dashed). Panels (**I**–**III**): Initial state and temperature as indicated at the bottom of each panel. Subplots (a–c) show comparisons of TCL2, TCL4, and P-mat, respectively, vs HEOM. Subplots (d) give the trace distance between HEOM and the aforementioned perturbative approaches (extending out to longer times). All other parameters are as in ref. 13 with cutoff frequency *γ*^−1^ = 50 fs. (**IV**) FMO dynamics comparison with a stronger coupling: Subplot (a) contrasts HEOM for cutoff frequency *γ*^−1^ = 166 fs against HEOM for *γ*^−1^ = 50 fs. Subplots (b,c) show P-mat and TCL2 (solid) against HEOM, all using cutoff frequency *γ*^−1^ = 166 fs. Notably, P-mat performs worse than TCL2 at short times but then improves. TCL4 provides unphysical solutions and has therefore not been included.

Panels (I–III) of Fig. 5 show the trace distances and populations from the weak-coupling techniques against the numerically exact HEOM calculations, all using *γ*^−1^ = 50 fs and for *T* = 77, 300 K as indicated. We find that TCL2 compares favourably to P-mat at short times, while for longer times, with the notable exception of Fig. 5(III), P-mat becomes more accurate. We suspect that this is because in the P-mat formalism, the secular approximation is inherently included. By contrast, in TCL a full or partial secular approximation^21^ still needs to be performed explicitly to guarantee that the system will go to the thermal state with respect to the temperature of the bath (provided there is no decoherence-free subspace^30^ and all the baths have a single temperature). Note, however, that in a stronger-coupling regime the system is no longer expected to fully evolve into its canonical thermal state at all^31^.

We conclude that in all these three cases, our weak-coupling techniques capture the relevant oscillations in the dynamics well, as expected. Rather surprisingly, in Fig. 5(III) (*γ*^−1^ = 50 fs and *T* = 300 K) when using site 1 as the initial state, TCL2 outperforms not only P-mat but also TCL4. We note that this is not the case in the same configuration but with site 6 as the location of the initial excitation.

We now consider the lower cutoff frequency *γ*^−1^ = 166 fs at *T* = 300 K, having already identified this case as one which violates our weak-coupling criterion. As shown in Fig. 5(IV), exact numerics suggest that the difference in the dynamics is deceptively small between *γ*^−1^ = 50 fs and 166 fs, borne out both by the populations [subplot (a)] and the trace distance [subplot (d)]. Nonetheless, according to Table 1, the two cases are vastly different from the point of view of the perturbation series, and indeed we find that TCL2 is overestimates the damping and does not capture the oscillations, whilst P-mat underestimates the damping and shows more oscillations than HEOM. Yet the main difference in this case is actually what is not shown in the figure: We do not present TCL4 results in this plot because in this regime, the TCL4 generator once more possesses an unphysical eigenvalue in the long-time limit. This means that, even though according to the trace-distance measure, TCL2 is not vastly different from Fig. 5(III) (*γ*^−1^ = 50 fs and *T* = 300 K), the 4th order of the perturbation is larger than the 2nd order in this case. This means that the 2nd order is neither justifiable nor trustworthy, and this is what our criterion captures.

## Discussion

We have introduced and discussed a straightforward criterion for the often difficult problem of deciding when when weak coupling approaches perform adequately for capturing higher-dimensional quantum dynamics in complex environments. We have discussed a rigorous as well as a simplified version of the criterion, with the latter constituting essentially a measure of the degree of ‘slippage of initial conditions’^26^,^27^.

By presenting a comparative numerical study of two different classes of weak-coupling methods, contrasted against numerically converged HEOM results, we have verified the validity and predictive power of both variants of the criterion. We have used the canonical spin boson model and the much-studied energy excitation dynamics in the FMO complex as two representative examples to make this case.

Interestingly, we have identified two room temperature FMO configurations with almost identical HEOM results, whilst our perturbative solutions diverge significantly, to the point that it yields un-physical results in the 4th order. This discrepancy is captured by our criterion which confirms that despite the apparent similarity of numerically converged results, one configuration sits well in the weak-coupling regime, whilst the other is sufficiently strongly coupled such that a perturbative approach is no longer justifiable. We note that, surprisingly, although not justified, it still gives fairly adequate results.

Finally, we have here extended the P-mat approach, first introduced in ref. 10, to a situation with multiple baths, and discussed its relationship to time-convolutionless master equations. We have seen that P-mat stands its ground reasonably well compared to TCL2, particularly in the long-time limit. Whilst not the subject of this study, we note that the concept of tiered environments^10^ can also be straightforwardly applied to higher-dimensional systems and multiple baths.

## Methods

The methods section of this paper is organised as follows: we first introduce the two aforementioned weak coupling approaches. We formally extend the P-mat technique from ref. 10 to a multisite system with independent bosonic baths. Following Breuer & Pettrucione^4^ we also briefly discuss the TCL technique. We then describe HEOM, which is our benchmark technique for numerically converged solutions.

### The Time Convolutionless (TCL) Technique

The TCL master equation is based on the projector-operator technique, stating that the system’s dynamics obeys the time-local master equation


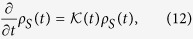


where the superoperator 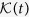 is known as the TCL generator, and *ρ*_*S*_(*t*) = Tr_*B*_(*ρ*) is the reduced density matrix of the system. It is time-local because *ρ*_*S*_ on the righthand side of Eq. (12) only features the current time *t*. All non-Markovian memory effects are thus contained within the TCL generator. A full derivation and discussion of it is found in chapter 9 of ref. 4.

Deriving an expression for the full TCL generator is as complex as solving the full von-Neumann equation for the system plus environment, so in practice we approximate it using a perturbative expansion in powers of the interaction:


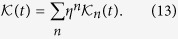


Note that this is sometimes not possible, but should be possible for short times/weak coupling^4^. We use the Hamiltonian (1), with a factorising initial condition *ρ*(0) = *ρ*_*S*_(0) ⊗ *ρ*_*B*_, where 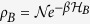, 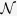 is the environment partition sum and *β* = 1/(*k*_*B*_*T*) inversely related to the temperature. For factorising initial condition and linear coupling, all odd terms in the TCL expansion vanish. The second and fourth terms are explicitly given by^4^,^32^:


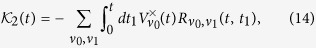



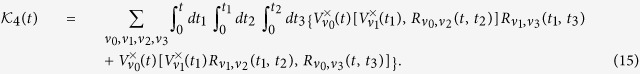


Here


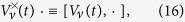



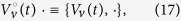



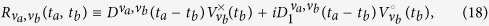


and 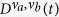, 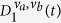 are the real and imaginary part of this response function, respectively, given by


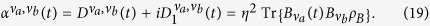


We note that in the strong-coupling regime, the procedure for calculating the TCL generator Eq. (13) may fail^4^. Even when it does not, there is no guarantee that a truncation will still yield physical results. Indeed, as discussed in the Results section we have found strong-coupling examples where TCL4 [i.e. truncating the series in Eq. (13) after the fourth order term] results in a positive, and thus unphysical, eigenvalue of the generator in the long-time limit.

### Multisite P-representation with Independent Baths

The P-matrix approach^10^ is a different weak-coupling expansion for approximating the reduced system dynamics. In this case, one approximates the *generating function* of the time evolution generator as opposed to approximating the *generator* like in the TCL technique. Interestingly, we find that this rather subtle distinction may have substantial impact even at second order.

In the P-matrix picture, we write down the dynamics of the reduced density matrix as


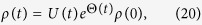


where *U*(*t*) is the evolution operator for the dynamics of the closed system, and the effects of the environment and its memory are captured by the influence functional Θ(*t*). Following ref. 10 we obtain a perturbative expansion of the influence functional in powers of the interaction parameter *η*, 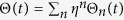. Interestingly, the influence functional expanded to second order bears a close formal relationship to the TCL expansion, and is simply given by


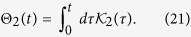


Note that in ref. 10 the P-matrix technique was developed for a single bath only, whereas Eq. (21) contains its extension to multiple independent baths.

### HEOM

The hierarchical equations of motion (HEOM), first proposed by Tanimura and Kubo^33^,^34^,^35^, map the exact equation of motion of the reduced system density matrix to a simpler set of equations describing a series of coupled auxiliary density matrices. This series is arranged in a hierarchy proven to converge. Derivation of this hierarchy requires that the response function of environment can be described by a sum of exponential terms. Examples of spectral densities where this is analytically the case include the Drude-Lorentz spectral density^33^, a Lorentzian spectral density^36^ and an underdamped Brownian oscillator^37^,^38^,^39^,^40^.

In the FMO example below we use a Drude-Lorentz spectral density. However, in general one can also numerically fit the response function of an arbitrary bath to a sum of exponential terms, similar in spirit to ref. 41. We find that this approach works well for both a Ohmic and super-Ohmic spectral densities. Due to the form of the HEOM, it is convenient to fit the real and imaginary parts of the response function independently, defining in general *α*(*t*) = *D*(*t*) + *iD*_1_(*t*), with


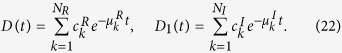


Then, when constructing the HEOM for such general spectral densities, we distinguish between those ancilliary density operators originating from the real and imaginary parts of the response function, and write the hierarchy index 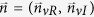. Here 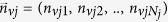, *n*_*νjk*_ are integers 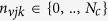, up to the cut-off tier of the hierarchy *N*_*c*_, and where 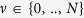 labels the different independent baths, up to their number *N*. For notational simplicity we assume all baths have the same correlation functions, but again generalisation is straight-forward. The HEOM (with renormalized coupling between hierarchies^42^) then takes the form


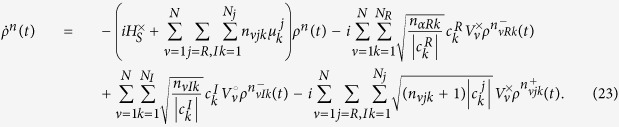


The notation 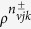 indicates an increase/decrease of just the *νjk*’th element of the hierarchy index by 1. One then solves these coupled equations numerically, setting all the ancillary 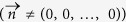 density matrices to zero at *t* = 0.

### Spin-Boson Response Function

In our simulation, the response function *α*(*t*) = *D*(*t*) + *iD*_1_(*t*) = 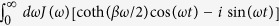 is approximated by a series of exponents, as given in Eq. (22). This was done by evaluating the exact response function at 0.1 ps intervals up to 8 ps, and using Mathematica’s “FindFit” function for non-linear least-squares fitting a function to a set of datapoints. Using this procedure we get the parameters given in Table 2, with the accuracy


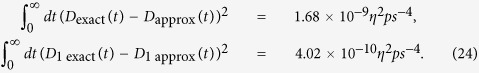


**Figure 6:**
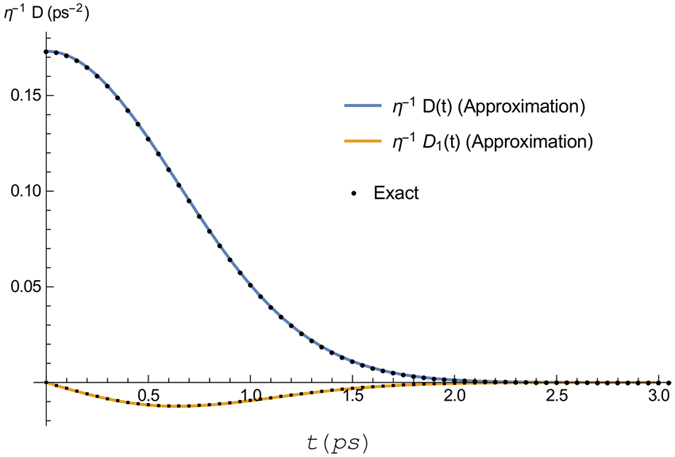
Exact and approximated response function, for an Ohmic bath characterised by a spectral density given by Eq. (9), with *T* = 50 *K*, *λ* = 0.01485 ps^−1^, and *ω*_*c*_ = 2.2 ps^−1^. The fitting parameters are given in Table 2.

In Fig. 6 we plot the exact and approximated response function in order to show the fit.

We find that for this case HEOM converges at *N*_*c*_ = 3 for *η* = 1 and *N*_*c*_ = 9 for *η* = 10.

### Derivation of the Criterion

In this section we derive the criterion (6) in the main text. For this analysis we look at the case where all of the different baths are independent, and they are all coupled to the system in the same manner, i.e. *J*_*ν*_(*ω*) = *J*(*ω*) for all *ν*. This simplifies Eqs (14 and 15) with 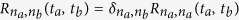, and 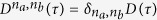, 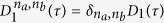, 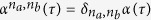.

Working in the Liouville Space 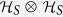, with |*i*〉 〈*j*|→|*ij*〉, where |*i*〉 are the system’s energy eigenstates, it is straightforward to write down an explicit expression for 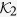:


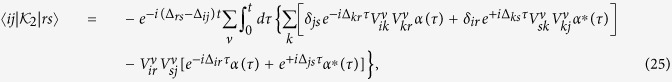


where Δ_*ij*_ = *ε*_*i*_ − *ε*_*j*_, and 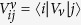.

The dephasing, decoherence, and Lamb-shift rates are given by matrix elements in the superoperator 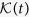 that do not oscillate with *t* as *t* → ∞, meaning that in 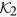 only elements with Δ_*rs*_ − Δ_*ij*_ = 0 contribute to the rates. In the same manner one can show that the *t* dependence of the fourth order is 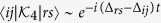, for times much longer than the bath’s memory time. Assuming that the energies are non-degenerate in the broad sense, meaning 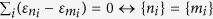, then the only matrix elements with these rates are either diagonal terms


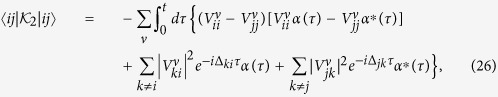


or elements between two different eigenstate projectors,


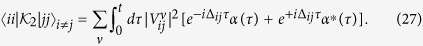


The part proportional to the diagonal of the coupling operator *V* is known in the literature as “pure dephasing”. Incidentally because our model only considers linear coupling between the system and environment, if there were only diagonal terms in the interaction, then the exact contributions of these terms are equal to their second order expansion^43^.

Moreover, we are only interested in the real parts of the rates, as the imaginary parts are responsible for the Lamb shift. Finally, in Eq. (26), the rates for *i* ≠ *j* are the ones for which the off-diagonal elements of the density matrix decay. For the sake of this paper we focus on the dephasing rates, i.e. the rates in which the diagonal elements of the density matrix decay, i.e.


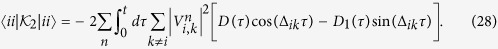


The part proportional to *D*_1_ = Im(*α*) in the above equations is normally much smaller and will be ignored for the purpose of our discussion, hence we approximate the second order rates as


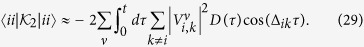


In the Markovian limit, where the time of the experiment is much longer than the memory-kernel decay time 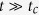, we may take the integral in Eq. (29) to infinity and obtain the usual rates


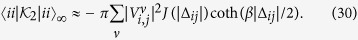


By contrast, for times comparable to or shorter than the decay time of the response kernel *D*(*t*), or if the spectral density is not smooth and has sharp peaks, it is not justified to take the Markovian limit and one should evaluate the rates (29) over the time of the experiment.

Using a similar analysis for the fourth-order terms, we find that corrections to the rates introduced by a fourth order treatment are given by


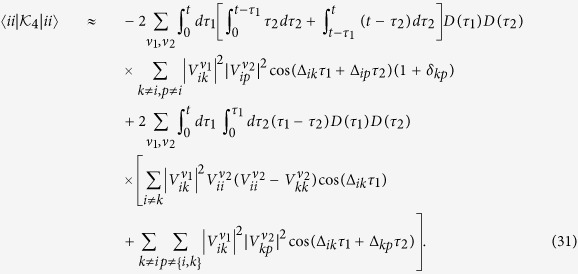


In the above equation we only kept terms that have *τ*_1_ or *τ*_2_ in their integrand. We note that there are other terms, namely terms with integrands similar to (*e*^−*i*Δ*τ*^ − 1)/Δ where Δ is an eigenfrequency of the system, which are generally, but not always, smaller than their *τ* equivalents derived by taking the limit Δ → 0. Further, we ignore contributions by *D*_1_ as in Eq. (29). Also, we assume that the system’s energies and frequencies are not degenerate in the sense that 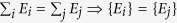.

The ratio between Eqs (29) and (31) defines our ‘full’ criterion [Eq. (5)]. To arrive at the ‘simplified’ criterion, we shall make some further approximations and assumptions in the following. We note that Eq. (31) consists of two terms and we conjecture that these are both of the same order (noting that this is a good approximation for for the spin-boson model and the FMO complex examples studied in this paper), so we continue with the first one for the sake of this analysis. Now, because we are looking for a criterion for when the fourth order contribution is small, we are being conservative by artificially enlarging it via the substitution 1 + *δ*_*kp*_ → 2, and taking the upper limit of the *τ*_2_ integral to *t*. Further, we expand the cosine and ignore any terms proportional to *τ*_2_ sin(Δ_*ik*_ *τ*_1_) sin(Δ_*ip*_ *τ*_2_), which are smaller than their cosine equivalents. Thus we arrive at


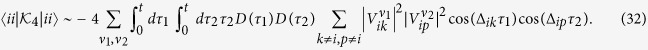


Now considering the ratio 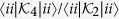 leaves us with the following ‘simplified’ criterion [Eq. (6)] for when weak-coupling is a good approximation:


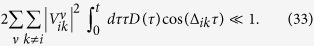


The above inequalities should hold for all of the system’s energy levels *i*.

### Raw Data

Raw data for all the entries of the density matrices described in this paper is included as a supplementary material. This includes HEOM, TCL2, TCL4, and P-mat data to for the Spin-Boson and FMO models plotted above.

## Additional Information

**How to cite this article**: Fruchtman, A. *et al*. When do perturbative approaches accurately capture the dynamics of complex quantum systems? *Sci. Rep.* **6**, 28204; doi: 10.1038/srep28204 (2016).

## Supplementary Material

2LS_TCL2_Strong.csv

2LS_HEOM_Strong.csv

2LS_TCL2_Weak.csv

2LS_Pmat_Weak.csv

2LS_Pmat_Strong.csv

2LS_HEOM_Weak.csv

2LS_TCL4_Weak.csv

FMO_TCL2_77KSite1.csv

FMO_TCL2_77KSite6.csv

FMO_TCL2_300KSite1.csv

FMO_TCL2_300K166Site1.csv

FMO_TCL4_77KSite1.csv

FMO_TCL4_77KSite6.csv

FMO_TCL4_300KSite1.csv

FMO_HEOM_77KSite1.csv

FMO_HEOM_77KSite6.csv

FMO_HEOM_300KSite1.csv

FMO_HEOM_300K166Site1.csv

FMO_pmat_77KSite1.csv

FMO_pmat_77KSite6.csv

FMO_pmat_300KSite1.csv

FMO_pmat_300K166Site1.csv

## Figures and Tables

**Table 1:** Full and simplifed criteria applied to different FMO configurations.

Temperature	*γ*^−1^	Max Full Criterion	Max Simplified Criterion	*λ*_*n*_/max |*V*_12_|
77 K	50 fs	0.04	0.09	0.4
300 K	50 fs	0.19	0.38	0.4
300 K	166 fs	1.09	2.6	0.4

In each case we choose the largest value of LHS of Eqs (5) and (6) over all *i*. The reorganisation energy, *λ*_*n*_ = 6.59 ps^−1^, does not depend on the cut-off frequency *γ* or the temperature, and we show the value of the reorganisation energy divided by the largest dipole-dipole coupling in the system, |*V*_12_| = 16.5 ps^−1^ (note that here 1 and 2 refer to the site basis, whereas throughout the rest of this paper we use the energy eigenbasis).

**Table 2:** Fitting parameters for a series of exponents form [Eq. (22)] of the response function of an Ohmic bath characterised by a spectral density given by Eq. (9), with *T* = 50 *K*, *λ* = 0.01485 ps^−1^, and *ω*_*c*_ = 2.2 ps^−1^.

Parameter	Value (*ps*^−2^)	Parameter	Value (*ps*^−1^)
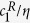	0.145 + 0.316*i*	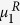	2.77 + 0.986*i*
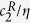	0.145 − 0.316*i*	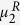	2.77 − 0.986*i*
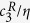	−0.0588 − 0.0207*i*	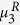	2.68 + 3.12*i*
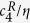	−0.0588 + 0.0207*i*	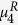	2.68 − 3.12*i*
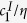	−0.00683 + 0.0449*i*	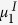	2.35 − 1.04*i*
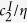	0.00683 + 0.00938*i*	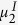	2.34 + 3.22*i*
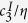	0.00683 − 0.00938*i*	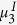	2.34 − 3.22*i*
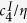	−0.00683 − 0.00449*i*	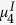	2.35 + 1.04*i*
